# Dissecting molecular mechanisms of immune microenvironment dysfunction in multiple myeloma and precursor conditions

**DOI:** 10.20517/2394-4722.2022.110

**Published:** 2023-05-16

**Authors:** Maria Moscvin, Benjamin Evans, Giada Bianchi

**Affiliations:** 1 Department of Medicine, Division of Hematology, Brigham and Womens Hospital, Boston, MA 02115, USA.; 2 Department of Medicine, Brigham and Women’s Hospital, Harvard Medical School, Boston, MA 02115, USA.; 3 Department of Medicine, Stanford University, Stanford, CA 94305, USA.

**Keywords:** Multiple myeloma, plasma cells, immune-microenvironment, immune response, immunotherapy

## Abstract

Multiple myeloma (MM) is a disease of clonally differentiated plasma cells. MM is almost always preceded by precursor conditions, monoclonal gammopathy of unknown significance (MGUS), and smoldering MM (SMM) through largely unknown molecular events. Genetic alterations of the malignant plasma cells play a critical role in patient clinical outcomes. Del(17p), t(4;14), and additional chromosomal alterations such as del(1p32), gain(1q) and MYC translocations are involved in active MM evolution. Interestingly, these genetic alterations appear strikingly similar in transformed plasma cell (PC) clones from MGUS, SMM, and MM stages. Recent studies show that effectors of the innate and adaptive immune response show marked dysfunction and skewing towards a tolerant environment that favors disease progression. The MM myeloid compartment is characterized by myeloid-derived suppressor cells (MDSCs), dendritic cells as well as M2-like phenotype macrophages that promote immune evasion. Major deregulations are found in the lymphoid compartment as well, with skewing towards immune tolerant Th17 and Treg and inhibition of CD8+ cytotoxic and CD4+ activated effector T cells. In summary, this review will provide an overview of the complex cross-talk between MM plasma cells and immune cells in the microenvironment and the molecular mechanisms promoting progression from precursor states to full-blown myeloma.

## INTRODUCTION

**Figure 1.**
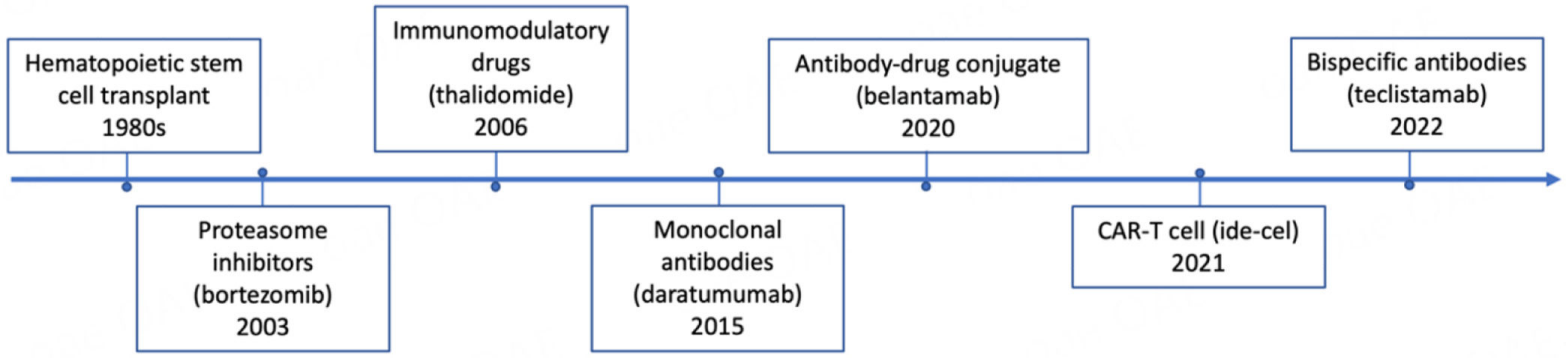
FDA-approved drugs for multiple myeloma and date of their first approval.

Multiple myeloma (MM) is a disease characterized by clonal expansion of terminally-differentiated plasma cells (PCs) in the bone marrow (BM)^[1]^. It is the second most common hematologic malignancy in the United States^[2]^. MM typically manifests clinically with end-organ damage consisting of anemia, renal impairment, lytic bone fractures, and hypercalcemia^[1]^. Over the past three decades, the introduction of novel treatments, such as proteasome inhibitors (PI), immunomodulatory drugs, autologous hematopoietic cell transplantation, and targeted monoclonal antibodies, has significantly improved the quality and length of life of patients with MM [Figure 1]^[3]^. However, de novo resistance has been reported, and acquired resistance is almost inevitable over time, contributing to the incurable nature of this disease^[4]^. Disease refractoriness is driven by tumor intrinsic and extrinsic factors. MM is characterized by genetic heterogeneity of malignant PC clones and therapy-induced clonal evolution may play a significant role in disease progression^[5–7]^. Extensive research has demonstrated that extrinsic factors such as a permissive immune microenvironment influence tumor cell behavior and disease outcome.

MM is almost always preceded by precursor conditions, monoclonal gammopathy of unknown significance (MGUS), and smoldering MM (SMM), through largely unknown molecular events^[8,9]^. Since premalignant states do not always progress to active myeloma, treatment is currently not justified solely on laboratory abnormalities, in the absence of symptoms. Genetic alterations of the malignant PC play a critical role in patient clinical outcomes. Del (17p), t(4;14), and additional chromosomal alterations such as del(1p32), gain (1q) and MYC translocations are involved in active MM evolution^[10]^. Interestingly, these genetic alterations appear strikingly similar in transformed PC clones from MGUS and SMM stages^[11,12]^. Emerging evidence indicates that tumors represent a complex ecosystem, and the combination of different conditions leads to a dynamic and self-fostering dysregulation of the immune system that supports tumor formation. Compositional and genetic expression changes of individual immune cell subtypes correlate with tumorigenesis and therapeutic outcomes^[13]^.

This review will navigate the immune system dysregulation observed in MM, exploring the molecular mechanisms and the dynamic cross-talk between the tumor and the microenvironment that is responsible for skewing immune cells towards tolerance.

## MYELOID COMPARTMENT

### Myeloid-derived suppressor cells

**Figure 2.**
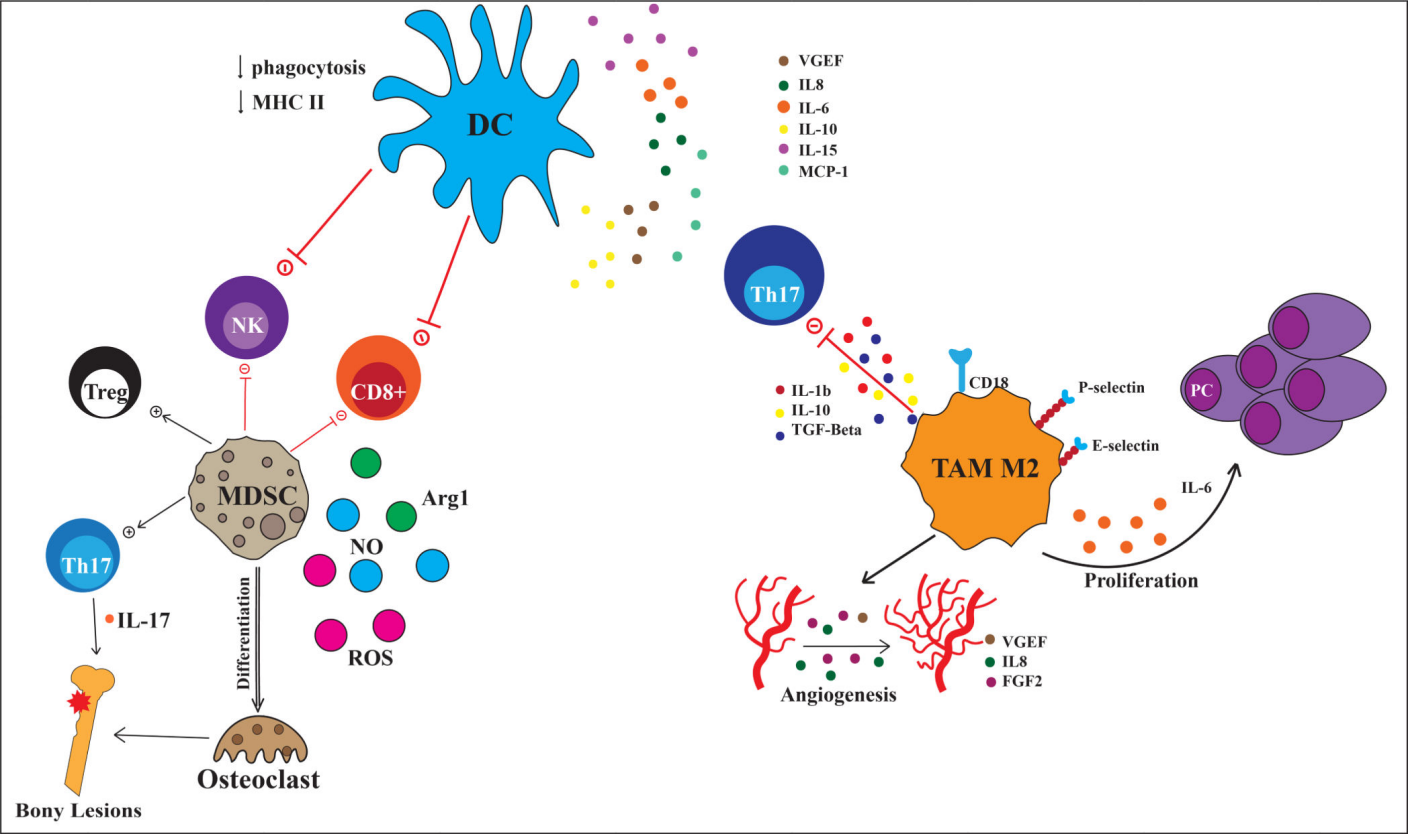
Myeloid cells in MM niche. Cartoon representing the cross-talk between myeloid derived suppressor cells (MDSC), dendritic cells (DC), Tumor-associated macrophages 2 (TAM M2) with MM plasma cells (PC) and Th17, T regulatory cells (Treg), Th17, cytotoxic CD8+ cells and natural killer cells (NK).

Myeloid-derived suppressor cells (MDSCs) are a heterogeneous population of myeloid cells in different stages of maturation. Previous studies have shown an increase in MDSCs in peripheral blood and BM of MM patients compared to healthy donors and MGUS patients^[14,15]^. In humans, MDSCs are commonly defined as the CD11b^+^CD33^+^HLADR^-/lo^ population in the mononucleated cells^[16]^. There are two main subsets identified based on the additional expression of surface markers: CD15 for granulocytic MDSC (g-MDSC) and CD14 for monocytic MDSC (Mo-MDSC)^[16,17]^. These cells have been extensively characterized in different cancer types, including MM. MDSCs secrete arginase, reactive oxygen species (ROS), and nitric oxide (NO), leading to the suppression of innate and adaptive immune responses and fostering tumor growth [Figure 2]. Through secretion of high levels of arginase, MDSCs induce depletion of L-arginine in the microenvironment, leading to T cell starvation^[18]^. Structurally, the depletion of arginine leads to impaired production of the CD3 chain which is an integral component of the T cell receptor (TCR)^[19]^. Further, inducible NOS (iNOS) plays an important role in the MDSC suppressive activity by synergistic interaction with Arginase-1 and generation of superoxide and NO^[20]^. NO mediates the suppression of the three inflammatory IL-2 receptor signaling pathways, as demonstrated by the lack of Jak2, STAT5, and Akt phosphorylation^[21]^. Besides the suppression of effector antitumor immunity, MDSCs have a stimulatory effect on tumor-promoting immune components by enhancing the expansion of regulatory T cells (Tregs) and T helper 17 (Th17) and inducing NK cell anergy [Figure 2]^[22]^.

In addition to the immunosuppressive functions, across different tumor models, including MM, MDSCs serve as osteoclast progenitors, suggesting a role of these cells in cancer-associated lytic bone lesions^[23,24]^. Zoledronic acid, a commonly used bisphosphonate, inhibits osteoclastogenesis and concomitantly decreases MDSCs in the BM^[23]^.

Daratumumab is a monoclonal antibody targeting CD38 and is now widely integrated into MM treatment combinations. Another mechanism of action through which this drug kills the myeloma cells is by creating an immunosuppressive environment through MDSCs depletion^[25,26]^. A recent study showed that CC motif chemokine ligand 5 (CCL5) and macrophage migration inhibitory factor (MIF) are molecules secreted by MM cells and have been found to be positive mediators of MDSC induction^[27]^. The same study found that IMiDs, such as lenalidomide and pomalidomide, decrease the secretion of CCL5 and MIF, resulting in suppressive effects on MDSC cells^[27]^.

### Neutrophils

Neutrophils are terminally differentiated cells that eliminate microbes and protect us against infections. However, cancer research on the role of tumor-associated neutrophils (TAN) has delivered controversial results^[28]^. Studies have shown that TANs possess both antitumor activities (e.g., direct cytotoxicity and inhibition of metastasis) and pro-tumor properties (e.g., promote angiogenesis, stimulate tumor cells migration and invasion, and support an immunosuppressive environment)^[28]^. Only in 2015, researchers found that neutrophils in cancer have a functional plasticity and can undergo “alternative activation” in response to signals from the tumor microenvironment. For example, the presence of transforming growth factor- TGF-) promote a pro-tumor phenotype (or N2 neutrophils), whereas interferon- INF) polarizes neutrophils towards an antitumor phenotype (or N1 neutrophils)^[28]^. Furthermore, multiple heterogeneous subsets have been observed. These subsets of neutrophils have the same immune phenotype but different patterns when undergoing density gradient centrifugation. Based on physical properties, these cells are now referred to as high-density neutrophils (HDNs) that sediment to the bottom, and low-density neutrophils (LDNs) that sediment at the top^[29]^. It is currently accepted that LDNs include g-MDSCs that have immunosuppressive properties, as described in the dedicated section. Additionally, the HDN subtype has been shown to have an N1-like phenotype and to kill tumor cells^[28,29]^.

Neutrophils from peripheral blood from MM patients have a different gene expression profile compared to those isolated from healthy donors or MGUS^[30]^. Compared to both healthy donors and MGUS, neutrophils from MM patients expressed dysregulated genes in several biological processes, including endocytosis, FC-R mediated phagocytosis, leukocyte trans-endothelial migration, and chemokine signaling in the Toll-like receptor pathways, and inositol-phosphate metabolism^[30]^.

The neutrophil-to-lymphocyte ratio (NLR) has been introduced as a prognostic factor for survival and response to treatment in many tumor types. In MM, the NLR can predict the outcomes at diagnosis or after treatment with novel agents as well as autologous stem cell transplantation^[30–32]^. A recent single-cell RNA sequencing article reported that only the frequency of mature neutrophils at diagnosis (and not other granulocytic progenitors) is significantly associated with patient outcome^[33]^. The same study showed that a high ratio of mature neutrophils/T cells at diagnosis is correlated with inferior progression-free survival (PFS)^[33]^.

### Monocytes and macrophages

Monocytes and macrophages constitute a heterogenous multi-functional cell population. Circulating monocytes are recruited into the tumor microenvironment (TME), where they are converted to tumor-associated macrophages (TAMs)^[34]^. Activated TAMs are generally classified into two types: an immunoreactive antitumoral M1 phenotype (classically activated) and an immunosuppressive pro-tumoral M2 phenotype (alternately activated). Fully polarized M1 and M2 macrophages are the extremes of a continuum of functional states^[35]^.

Macrophages heavily infiltrate the BM of myeloma patients relative to the BM of healthy controls^[36,37]^. Myeloma-associated macrophages (MAMs) in the BM niche are predominantly skewed phenotypically and functionally toward M2 phenotype^[36,37]^. MAMs provide nurturing signals to MM cells, promote immune escape, and negatively correlate with patient survival^[38]^. Interactions between integrins on MAMs and MM cells induce Src, Erk1/2 kinases, and c-Myc pathways, suppressing caspase activation and supporting tumor cell survival^[39]^. Besides contact mechanisms, human MAMs constitute a relevant source of pro-tumoral interleukin-1b IL-1), IL-10, IL-6, and tumor necrosis factor-alfa [Figure 2]^[40]^.

Importantly, macrophages also support MM progression through direct and indirect action on MM-associated neo-angiogenesis. Studies have found that angiogenesis is differently regulated in myeloma compared to precursor stages, suggesting that dysregulation in angiogenic cytokines and cells as well as hypoxia may contribute to myeloma progression^[41–43]^. Endothelial cells (EC) and epidermal growth factor receptor (EGFR) signaling have been shown to promote bone marrow angiogenesis and disease progression in preclinical models^[42]^. Interestingly, myeloma macrophages also secrete proangiogenic factors such as vascular endothelial growth factor (VEGF), IL-8, and fibroblast growth factor-2 (FGF-2)^[44]^. Macrophages derived from MM patients exposed to VEGF and FGF also show vasculogenic mimicry by acquiring endothelial cell markers and generating capillary-like vessels, in contrast with macrophages from normal subjects or MGUS^[44]^. Agents that block the VEGF signaling normalize the vasculature, improving oxygenation and delivery of chemotherapies to tumor cells while limiting the perfusion of the hyper-vascularized tumor areas. Clinical trials in MM tested anti-angiogenic agents such as bevacizumab used in combination with other MM drugs^[45–47]^.

Several studies report that MAMs protect myeloma cells from chemotherapy-induced apoptosis, thereby contributing to melphalan and bortezomib resistance^[37,48]^. Recent studies show that the IKZF1 IRF4/IRF5 axis is relevant to drive M2 pro-tumoral skewing^[49]^. Interestingly, lenalidomide, which is an immunomodulatory drug commonly used in MM, induces cytotoxicity partially through inhibition of this axis^[50]^.

Notably, therapeutic approaches aimed at depleting, inhibiting, or reprogramming macrophages have shown promising results in seminal preclinical cancer models. Currently, a common approach to induce macrophage depletion involves targeting the CSF-1 receptor (CSF1R), an important mediator of macrophage survival and differentiation. A recent study has demonstrated that anti-CSF1R antibody reduces tumor burden and improves survival in MM preclinical models^[51]^. In line with the unique plasticity of macrophages, MAMs retain their tumoricidal potential, making macrophage-repolarization an intriguing therapeutic strategy^[52]^. Current evidence from preclinical models shows that exposing MAMs to a cocktail of cytokines promoting M1 and contrasting M2 phenotype mediate M1-reprogramming^[53]^. An alternative strategy to achieve a similar result involves blocking the immune checkpoint CD47, a “don’t eat me” signal expressed on MM cells^[54]^. One study showed that Ruxolitinib, a Jak1/Jak2 inhibitor, induces an increase in the M1/M2 ratio in myeloma, suggesting that skewing towards a tumor-suppressive M1 phenotype is feasible^[55]^.

### Dendritic cells

Dendritic cells (DCs) are antigen-presenting cells (APCs) that play prominent roles in mediating both innate and adaptive immune responses^[56]^. DCs derive from BM mononuclear cells and are classified based on developmental stage into immature and mature DCs^[57]^. During the maturation process, DCs acquire HLA-DR expression, costimulatory molecules such as CD80, CD86 and generate specific T/B cell responses through the so-called cross-presentation process^[58]^. DC are further classified according to their origin, phenotype, and function in myeloid DCs (mDCs) and plasmacytoid DCs (pDCs). Evidence about the role of DCs in MM pathogenesis is controversial and the mechanism by which DCs contribute to the immunosuppressive microenvironment is not fully uncovered. pDCs interaction with MM cells stimulates the secretion of soluble factors, such as IL-10, VEGF, IL-8, IL-15, MCP-1, and IL-6, in the BM niche [Figure 2]^[59]^. This contributes to the deregulation of the phagocytosis and processing and presentation of antigens with a concomitant reduction in the expression of costimulatory molecules^[60–62]^. Other relevant pro-survival cytokines include BAFF (B-cell activating factor) and APRIL (a proliferation-inducing ligand) that bind to BCMA (B-cell maturation antigen) on MM cells, promoting proliferation via NF-B and MAPK. The pDCs stimulatory effect can be abrogated by disrupting the NF-B pathway through the inhibition of BAFF/APRIL binding to BCMA^[63]^.

Our group showed an increase in pDCs accumulation in the BM of MM patients compared to healthy donors^[62]^. We also reported that pDCs derived from the BM of MM patients have a decreased capacity to stimulate T cell response, support growth of MM, and contribute to drug resistance through secretion of cytokines such as SDF-1 (CXCL12) and IL-3^[62]^.

However, the function of DCs in MM patients is still controversial. One group suggested that, based on the viability of MM cells, BM DCs play a dual and opposing role. Specifically, apoptotic malignant plasma cells undergo phagocytosis by bone marrow mDCs and pDCs, leading to the generation of tumor-specific cytotoxic T cells. However, the interaction of mDCs with nonapoptotic tumor plasma cells induces evasion from human leukocyte antigen (HLA) class I-mediated CD8 T cell killing by downregulating the synthesis of proteasome subunits in these cells and processing of antigens^[64]^. In this context, it has been shown that MM-derived pDCs and MDSCs express high levels of cell surface programmed-death-ligand 1 (PD-L1). The binding of PD-L1 to its receptor, programmed death-1 (PD-1), activates downstream signaling pathways and triggers apoptosis and anergy of T and NK effector cells, conferring a cancer immune-tolerant environment^[62,65]^.

Genomic instability is an increased tendency to acquire genomic alterations and is one of the hallmarks of myeloma, both at early and advanced stages^[66]^. Interestingly, not only do the DCs support tumor growth, but they also regulate the genomic integrity of MM cells. Koduru *et al*. reported that the interaction between myeloma and DCs leads to rapid induction of the activation-induced cytidine deaminase (AID) enzyme and AID-dependent double-strand DNA breaks in myeloma cell lines as well as primary MM cells^[67]^.

Further, DCs may be implicated in the development of osteolytic lesions in MM patients. It has been shown that DCs induce the expansion of polyfunctional Th17 in the BM, followed by IL-17 secretion, a potent pro-osteoclastogenic factor^[68]^.

These data suggest that DCs may directly impact the biology of MM. Based on these premises, several drugs targeting DCs-MM interaction are under investigation. The inhibition of the CD28/CD80/CD86 axis with CTLA4 inhibitors is currently being explored in MM patients^[68,69]^. Additionally, pDCs express high levels of CD38 and daratumumab has been shown to cause depletion of pDCs^[70]^.

## LYMPHOID COMPARTMENT

### Tregs and Th17

**Figure 3.**
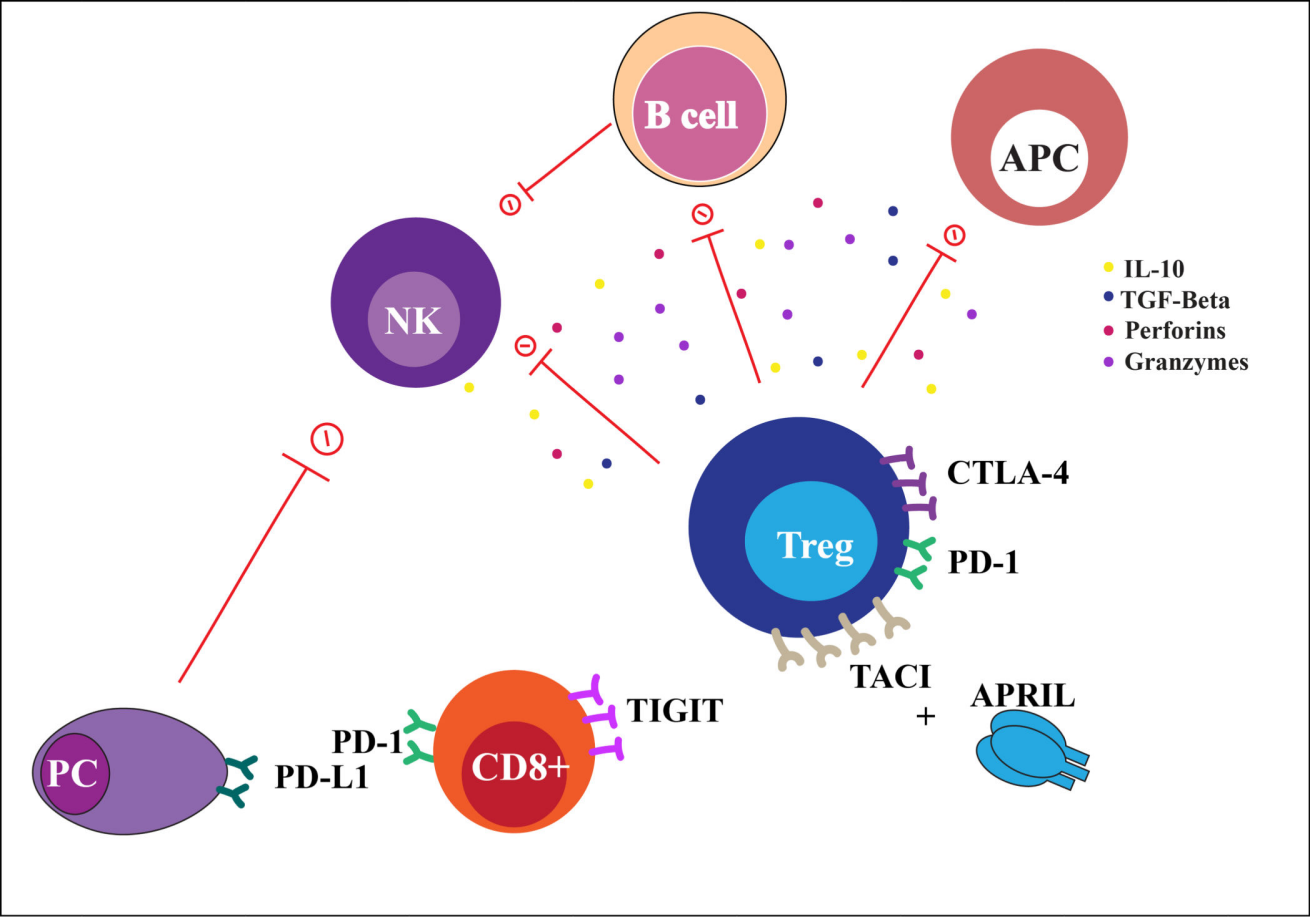
Lymphoid cells in multiple myeloma niche. Cartoon representing the interaction between lymphoid compartment and MM plasma cells (PC). Cytotoxic T cells CD8+ and regulatory T cells (Treg) in multiple myeloma have increased expression of immunosuppression molecules, with suppression of B cells, APC and NK cells activity.

Naïve CD4+ T lymphocytes differentiate into T helper 1 (Th1), Th2, Th17, and regulatory T cells (Tregs) depending on the combination of cytokines in the microenvironment [Figure 3]. The complexity of T-cell immunity has been extensively investigated in myeloma and precursor diseases. Abnormalities in the function and distribution of T cell subsets have been reported in active MM, including expansion of Tregs and pro-inflammatory Th17, reduced Th1/Th2 ratio cytokine production, and altered stem-like capacity of the T cell compartment^[68,71,72]^.

Tregs are a specialized subset of CD4+ T cells that have been associated with immune evasion in cancers. Tregs suppress the function of APCs, B cells, NK cells, and tumor-specific effector T cells by direct cellular interaction or by secretion of anti-inflammatory cytokines (e.g., IL-10 and TGF- β) and cytolytic granules (e.g., granzymes, perforins)^[73,74]^. An expansion of Tregs in the peripheral blood has a negative impact on survival and has been associated with a higher tumor burden in myeloma^[72,75]^. Recently, a preclinical study showed that in vivo depletion of Tregs in a MM murine model evokes a potent CD8+ T cell- and NK cell-mediated immune response, resulting in tumor regression^[76]^.

Similar to pDCs and MDSCs, the PD-1/PD-L1 axis has also been studied in MM Tregs. Co-culture of CD4+ T cells with MM cells results in the generation of functional Tregs in a contact-dependent and antigen-presenting cell-independent manner. These Tregs show increased PD-1 expression compared to naturally occurring Tregs^[77]^. Preclinical studies have shown greater PD-L1 expression on myeloma plasma cells compared to MGUS or healthy donor plasma cells, contributing to immune escape mechanisms^[78–80]^.

APRIL is a ligand for both BCMA and TACI (transmembrane activator calcium modulator and cyclophilin ligand interactor) receptors. Preclinical studies show that TACI is highly expressed in Tregs of MM patients and APRIL serum levels are increased in MM patients [Figure 3]^[81]^. APRIL increases MM-driven Tregs via TACI-dependent proliferation associated with upregulation of immunosuppressive cytokines, such as IL-10, TGF β−1, and CD15s^[81]^. Additionally, APRIL binds BCMA receptor and is highly secreted by MM-derived osteoclasts, potentially contributing to myeloma bone disease^[82]^.

The therapeutic implications of Tregs are numerous. The immunomodulatory activity of lenalidomide is partly driven by the downregulation of inducible T-cell costimulatory ligands, a decrease in Treg population, and their respective FoxP3 expression^[83,84]^. Recently, a novel subpopulation of CD38-positive Tregs was identified. *In vitro* studies have shown that this subset of Tregs has a more potent immunosuppressive activity compared to CD38 negative Tregs and is significantly reduced in daratumumab-treated patients^[25]^.

The Treg/Th17 balance in the microenvironment of MM patients is considered to be a marker of immunoregulatory control^[85]^. Physiologically, Th17 are pro-inflammatory cells and secrete among others, IL-17, IL-6, IL-22, and TNF-α cytokines. Seminal preclinical studies provide evidence that supports the pivotal role of Th17 and IL-17 in myeloma development and progression. MM patients show a significant imbalance in Treg/Th17 ratio when compared to either healthy donors or other monoclonal gammopathies and this correlates with worse long-term survival^[86–88]^. Clinically, the proportion of Th17 cells in the bone marrow positively correlates with tumor stage, serum lactate dehydrogenase, and serum creatinine concentration^[87]^. Preclinical studies reported an association between Th17 and MM cell proliferation, migration, neoangiogenesis, immune evasion, and myeloma bone disease^[68,89]^. Consistently, Noonan *et al*. showed that Th17 are enriched in the BM, where they mediate the development of lytic bone lesions via secretion of IL-17^[88]^.

### Cytotoxic T cells

CD8+ T cells are effector lymphocytes characterized by cytotoxic tumor-specific activity. Interestingly, CD8+ T cells are equally prominent in precursor conditions, active myeloma, and healthy donors. However, in MM, BM cytotoxic T cells have an altered capability to respond to tumor-specific antigens. Several mechanisms have been proposed to corroborate this immune evasion, including the ineffective antigen presentation capacity of the dendritic cells and a protective myeloid compartment^[33,90–92]^. Supporting this rationale, a preclinical study showed that T cells from patients with clinically progressive myeloma were found to induce a potent cytolytic activity against freshly isolated autologous tumor cells, only after *ex vivo* stimulation with autologous dendritic cells^[93]^. Subsequent studies showed that dendritic cells primed with myeloma cell lysates induce a potent tumor-specific cytotoxic T cell response^[94]^. These data provide evidence that endogenous cytotoxic T cells have the potential to be activated to elicit an anti-MM response.

MM and tumor microenvironment cells secrete IL-10, TGF-β, immunosuppressive ectoenzymes and other soluble factors, which potentially modulate the cytotoxic activity of CD8+ cells^[95]^. The ectoenzyme family relies on adenosine, which is a well-characterized immunosuppressive metabolite^[96]^. In the extracellular space, ATP is metabolized to adenosine by the sequential activity of CD39 and CD73, which are two extracellular enzymes. Specifically, CD39 converts ATP and ADP to AMP, and CD73 rapidly metabolizes AMP to adenosine^[96,97]^. MM cells express high levels of CD38 and CD39 surface molecules. Consistently, higher levels of adenosine are detected in the serum of patients with active myeloma compared to patients with precursor myeloma disorders and healthy donors^[98–100]^. Similarly, a recent study using a murine model of MM showed that inhibitors of the adenosine pathway induce activation of immune cells, increase interferon-gamma production and reduce myeloma tumor load^[99]^.

Studies have shown that active myeloma is characterized by a dynamic alteration of CD8+ phenotype ranging from “senescent” to “exhausted”. CD8+ T cells express molecules associated with T cell exhaustion (PD-1, CTLA-4, CD160, 2B4, LAG3) and T cell senescence (CD57, KLRG-1, lack of CD28) [Figure 3]. Unfortunately, recent trials showed disappointing clinical benefits of anti-PD-1 blockade therapy in myeloma^[101,102]^. Importantly, TIGIT (T cell Immunoreceptor with immunoglobulin and ITIM domains) has recently emerged as a promising immune checkpoint in MM. High levels of TIGIT expression on CD8+ T cells strongly correlate with MM progression, both in mice and humans^[103]^. TIGIT+ T cells originating from MM patients are characterized by decreased proliferation and inability to secrete cytokines in response to myeloma antigen stimulation [Figure 3]^[103]^. Monoclonal antibodies blocking TIGIT increase the effector function of CD8+ T cells and suppress MM development, reverting the dysfunctional phenotype^[104,105]^. Notably, TIGIT inhibition induces a reduction in tumor burden, prolonged survival in preclinical Vk*MYC MM models and prevents immune escape in myeloma murine models undergoing stem cell transplant^[103,105]^.

### NK cells

NK cells are small granular lymphoid cells exerting cytotoxic activity against tumor cells. While studies reported an increase in NK cells in the peripheral blood and BM of MM patients, other studies revealed a decrease in this population^[106–108]^. Importantly, NK activity is impaired, especially in cases of clinically advanced MM disease^[109,110]^. To discriminate between target and healthy cells, NK cells express surface receptors that induce cytotoxic activation (such as NKG2D, NCR, DNAM-1, CD16) or inhibition (such as KIR, CD94/NKG2A)^[111]^. MICA is a well-known ligand present on tumor cells that binds the receptor NKG2D on NK cells. Studies have shown that as disease progresses, MICA is shed from the surface of MM cells and NKG2D is internalized, impairing NK cell activation and killing of the tumor cell^[112–114]^. Similarly, DNAM-1 expression is reduced as MM progresses while its ligand PVR is upregulated^[114]^. Interestingly, IL-6 has been associated with down-regulation of perforin expression through NF-kB and STAT3 pathways, potentially contributing to impaired NK cell cytotoxicity^[115,116]^. A decrease in NK cell surveillance and cytotoxicity against MM might also be partially driven by the up-regulation in the expression of PD-1 on NK cell surface, which accompanies the increase in PD-L1 expression on MM cells^[117]^. However, inhibitors of the PD-1/PD-L1 pathway have been ineffective as single agents in MM^[118]^.

In patients with long-term disease, autologous stem cell transplantation induces an increase in NK population. Recent clinical trials have focused on boosting NK-related immunosurveillance via activation and expansion of NK cells *ex vivo* or by using allogeneic cord blood-derived NK cells^[119,120]^. A strategy to activate and expand functional NK cells is based on using engineered cells expressing ligands that induce NK activation combined with a cocktail of specific cytokines. Based on these biological premises, a recent seminal protocol for cytokine-induced memory-like (CIML) NK cell development was established^[121]^. Specifically, NK cells undergo *ex vivo* pre-activation with IL-12, IL-15, and IL-18 before administration to patients^[121]^. Our group is currently conducting a clinical trial employing the use of CIML NK cells along with low-dose IL-2 in newly diagnosed MM patients (NCT04634435)^[121]^.

## INFLAMMATORY MESENCHYMAL STROMAL CELLS

Mounting evidence from preclinical models reproducing MM cells in the microenvironment niche suggests that mesenchymal stromal cells (MSCs) support MM development and induce drug resistance and immunomodulation via direct cellular interaction and soluble factors^[122,123]^. A recent single-cell RNA sequencing study comprehensibly characterized an inflammatory phenotype of MSCs (iMSCs) nearly exclusive to the MM microenvironment^[124]^. The investigators proposed a model whereby soluble factors such as IL-1 secreted by monocytes and TNF secreted by NK and CD8+ lymphocytes promote the inflammatory phenotype in stromal cells. They also speculated that tumor cells present in the BM induce inflammatory MSC phenotype by activation of immune cells leading to the production of inflammatory cytokines and by releasing exosomes containing DAMPs.

**Figure 4.**
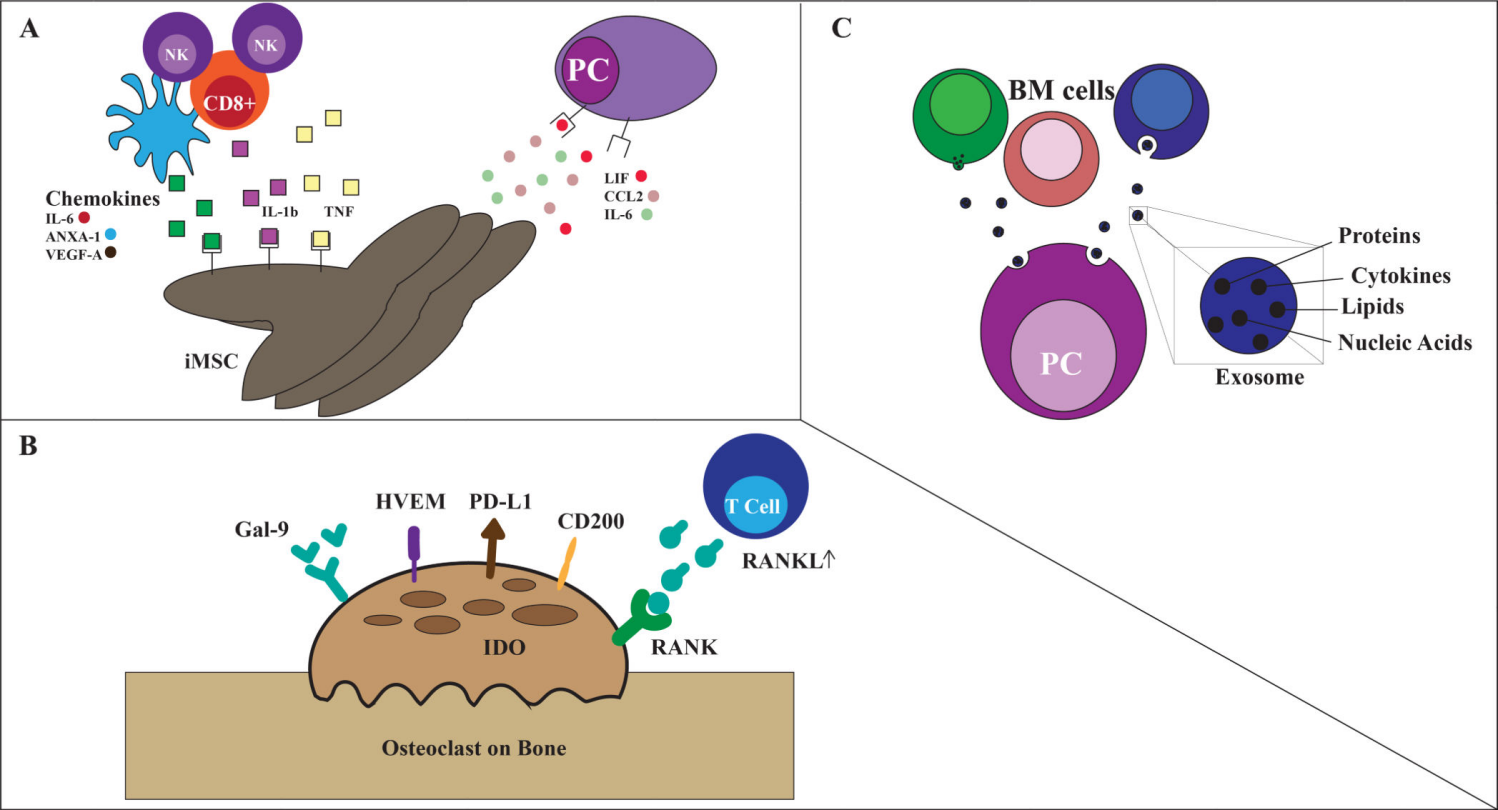
Other microenvironment components. (A) Inflammatory mesenchymal stromal cells (iMSCs): IL-1, TNF-alfa secreted by myeloid cells, cytotoxic T cells and NK cells and DAMPs deriving from tumor cells induce inflammatory phenotype of stromal cells. In turn, iMSC support tumor development through secretion of IL-6, LIF, CCL2 among other cytokines. iMSC simultaneously recruit and modulate immune cells, primarily myeloid cells. (B) osteoclasts: upregulation of Gal-9, CD200, HVEM, CD200, PD-L1, RANK in osteoclasts with secretion of IDO induce immuno-suppression; osteoclasts also produce OPN, IL-6, BAFF and APRIL that support MM survival. (C) Exosomes: BM cells in MM compared to healthy donors showed lower levels of miRNA-15a and increased levels of pro-tumoral Il-6, fibronectin, and CCL-2.

In turn, iMSC secrete IL-6, LIF, and CCL2 that support tumor cell proliferation and IL-6, C3, ANXA-1, and VEGFa that modulate immune cell compartment, particularly myeloid cells [Figure 4A]^[124]^.

## OSTEOCLASTS AS IMMUNOCOMPETENT CELLS

The imbalance between bone deposition and bone resorption is responsible for osteolytic bone lesions, a hallmark of myeloma development. Besides their function on bone metabolism, osteoclasts (OCLs) have an immunosuppressive and pro-tumoral role in the MM BM microenvironment. Preclinical studies show that OCLs produce MM pro-survival factors, such as osteopontin (OPN), IL-6, BAFF, and APRIL [Figure 4B]^[82,125]^. Studies have shown that OCLs significantly protect MM cells against T-cell mediated cytotoxicity via direct inhibition of proliferating CD4+ and CD8+ T cells^[126]^. The immunosuppressive effect is mediated by the up-regulation of immune checkpoint molecules including PD-L1, Galectin-9, CD200, herpesvirus entry mediator (HVEM), and secretion of high levels of indoleamine-2,3-dioxygenase (IDO), a metabolic enzyme leading to suppression of T-cell function [Figure 4B]^[126]^.

OCLs inhibit T cells, which in turn enhance osteoclastogenesis. In fact, in MM co-culture systems, activated T lymphocytes secrete high levels of RANKL, the main pro-osteoclastogenic factor^[127]^. Consistently, MM patients with osteolytic lesions show RANKL up-regulation by BM T cells as compared to MM patients without bone lesions^[127]^. Lastly, MM T cells secrete IL-3 that promotes MM-induced osteoclastogenesis and levels of this cytokine are higher in MM patients compared to controls^[128]^.

As described in previous paragraphs, MM patients show an increase in IL-17-producing Th17 that inhibits cytotoxic T-cell activity and promotes MM cell growth^[68]^. Interestingly, levels of cytokines that selectively induce Th17 phenotype tightly correlate with lytic bone lesions^[68,88]^.

## EXOSOMES

Exosomes are 30–100 nm small, secreted vesicles containing nucleic acids, proteins, and lipids. They are generated via multivesicular endosomes and are subsequently released upon fusion of these endosomes with the cell membrane [Figure 4C]^[129–131]^. Recruitment and clustering of macromolecules generally occur either via endosomal sorting complex required for transport (ESCRT)-dependent or ESCRT-independent mechanisms^[131,132]^. Once secreted, exosomes serve as vital cross-talking mediators between the bone marrow microenvironment and surrounding MM^[133,134]^. In doing so, they promote angiogenesis, osteolysis, and drug resistance, contributing to MM progression^[135]^. The interaction of exosomes with surrounding cells occurs through distinct mechanisms, primarily mediated by the direct fusion with the plasma membrane, or incorporation via pathways such as phagocytosis. Upon release of exosome content, downstream intracellular signaling is subsequently activated^[131]^. Exosomes derived from MM cells have the capacity to reprogram cells in the bone marrow to promote a pro-tumor environment that supports disease progression^[136]^. This can be attributed to elements such as cell recruitment, immunosuppressive effects, and horizontal transfer of genetic information^[133,134]^. *In vitro* studies have demonstrated that exosomes derived from the bone marrow stromal cells (BMSCs) induce MM growth, survival, and drug resistance and subsequent disease progression^[137]^. Preclinical studies showed that exosomes secreted by BMSCs derived from MM patients promote tumor growth in contrast to exosomes derived from healthy patients that have demonstrated opposing effects^[138,139]^.

In-depth profiling of the BMSCs exosomes content in MM compared to healthy donors, demonstrated lower levels of the tumor-suppressive factor miRNA-15a, and higher levels of pro-tumoral molecules such as chemokine C-C motif ligand (CCL) 2, IL-6, and fibronectin [Figure 4C]^[139]^.

Exosomes can also induce drug resistance mechanisms via inter-cellular transfer of molecules. In fact, it has been shown that exosomes containing specific molecules such as PSMA3 and PSMA3 Antisense RNA1 were transferred from PI-resistant MM patients to sensitive MM patients, inducing proteasome inhibitor resistance by increasing the proteasome activity^[140]^.

Adding to the complexity of the system, the interaction between micro-environmental cells and MM cells dictates the composition of the exosomes in the ecosystem. Our recent work showed that co-culture of MM with BMSCs cells induces HDAC3 expression in BMSC cells, while HDAC3 knockdown in BMSC leads to quantitative and qualitative changes in secreted exosomes that ultimately contribute to MM cell growth arrest[141].

Within the bone marrow microenvironment, exosomes contribute to a pro-osteoclast microenvironment through non-coding RNAs (ncRNAs)^[142,143]^. Generally, high osteoclasts to osteoblasts ratio induces bone reabsorption and myeloma bone disease. Certain pathways have demonstrated increased osteoclastogenesis. Seminal studies have shown that molecules enriched in MM-exosomes such as lncRNA RUNX2 antisense RNA 1 (RUNX2-AS1), amphiregulin, and miR-129–5p increase osteoclastogenesis by reducing RUNX2 splicing efficiency, activating the epidermal EGFR pathway, and downregulating the expression of the transcription factor Sp1, respectively^[144–146]^. Exosomes have also shown the ability to downregulate osteoblastogenesis through the suppression of osteoblastic differentiation proteins such as Runt-related transcription factor 2 (Runx2), Osterix, and osteocalcin^[145–147]^.

## IMMUNE MICROENVIRONMENT MODULATION IN PROGRESSION FROM PRECURSOR STAGES TO ACTIVE MYELOMA

In recent years, genomic studies provided the opportunity to dissect the genetic alterations that occur in active myeloma and precursor stages with unprecedented accuracy. It has been shown that MGUS/SMM patients may already harbor chromosomal alterations that define MM. However, no driver genetic mutation has been identified to date; hence, the cause of MM pathogenesis and progression from MGUS/SMM/MM remains elusive. Importantly, dissecting the mechanisms of evolution of the immune microenvironment from precursor non-malignant stages to active myeloma could pave the way to develop strategies for immune-based patient stratification and therapeutic strategies aiming at delaying this progression and potentially eradicating MM.

A recent single-cell study investigating the cellular composition of the tumor microenvironment reported a significant, although heterogenous, enrichment of T cells, CD16+ monocytes, and NK cells at the MGUS stage^[13]^. It has been shown that mature CD14+ monocytes are already dysfunctional at the MGUS stage, presenting a phenotypic shift leading to loss of major histocompatibility complex class II (MHC II) expression. Therefore, CD14+ monocytes have an impairment of their antigen-presenting cell capacity with suppression of the T cell activation, as early as in the MGUS stage^[13]^. Studies on matched samples showed an expansion of monocytes and macrophages during the progression from SMM to full-blown MM^[148]^. Similarly, Calcinotto *et al*. showed that patients with active MM, compared to MGUS/SMM, present an expansion of macrophages in the BM microenvironment that associates with increased BM vascularity and poor prognosis^[149]^. The same group suggested that the progression from MGUS/SMM to MM is also driven by an “angiogenic switch” characterized by an increase in BM plasma levels of angiogenic cytokines^[149]^. This was further confirmed by a large prospective study that showed that a composite angiogenesis biomarker score, calculated based on the levels of EGF, HGF, and Ang-2, correlated with an increased risk of MGUS progression to MM^[150]^.

In the context of APC cells, although researchers have speculated that the clinical progression from MGUS to MM may be driven by defects in the dendritic cell function, evidence supporting this assumption is controversial. While certain studies reported that mDCs and pDCs accumulate in the BM during MGUS to MM progression, others have shown a significant depletion of both circulating and BM pDCs in patients with MGUS and active MM, compared to healthy donors^[64,151]^.

Seminal work has been done to extensively characterize the T-cell compartment and its potential role in myeloma development from precursor stages. *Ex vivo* T-cells derived from BM of patients with preneoplastic gammopathy retain a vigorous antitumor activity against premalignant plasma cells^[152]^. This is in contrast to T cells from myeloma bone marrow, which lack tumor-specific rapid effector function, suggesting that T cells in MM lose the ability to naturally control tumor progression^[152]^. Studies on the Vk* MYC MM mice model showed an accumulation of CD3+ T cells, both CD8+ and CD4+ T cells, during disease progression^[149]^. Analysis of the cytokine composition of CD4+ T cells revealed a progressive loss of Th1 immune response and skewing toward Th2 response in Vk*MYC compared to WT mice^[149]^. Conversely, a recent single-cell study showed a depletion of CD4+ lymphocytes and a heterogenous pattern of expression of CD8+ cells during the progression from SMM to MM in matched samples^[148]^.

Among the immune system dysfunctions identified during the progression to MM, it is worthwhile mentioning the increased immunosuppressive T phenotype starting from the MGUS stage. Studies reported an expansion of immunosuppressive Tregs and T-cell exhaustion phenotype, starting from MGUS, suggesting that T-cell dysfunction might be an early event^[13,72]^. Th17 cells and gut microbiota might also play a role in the progression. A recent study on Vk*MYC mice showed that gut microbiota promote the differentiation into Th17 cells, which migrate to the BM, where they favor the progression from SMM to MM[153].

Lastly, changes in the NK cell compartment in the BM microenvironment have been reported in the progression of MGUS to MM^[154]^. In a recent single-cell study of matched samples, investigators have reported a highly dynamic microenvironmental NK profile over patients’ disease course, resulting in the arduous interpretation of a common trend for how tumor microenvironment evolves during disease progression^[148]^. Liu *et al*. reported that in the NK population, CXCR4-expressing NK was prevalent during active disease, while CX3CR1-expressing NK was more intensively represented post-transplant^[148]^. Similarly, another single-cell study by Zavidij *et al*. showed that in patients with high NK-cell infiltration, this fraction was predominantly constituted of CXCR4+ cells, while patients with fewer NK cells showed a shift toward CX3CR1 expression^[13]^. Importantly, previous studies have shown that these are chemoattractant receptors responsible for mediating homing to the BM^[155,156]^. This may explain the heterogenous representation of NK subsets observed in these studies and could suggest an MM-orchestrated mechanism of immune evasion.

## IMMUNOTHERAPY IN MULTIPLE MYELOMA

Immunotherapy is a type of cancer treatment that boosts the immune system to recognize and kill tumor cells. In the past few decades, the understanding of the immune system composition and the better characterization of myeloma antigens have been instrumental in developing immunotherapies for the treatment of MM. Immune-based treatment approaches are gaining supremacy over traditional therapies not only in myeloma but also in other liquid and solid tumors. This can be explained by high efficiency and specificity, with a more manageable toxicity profile. Novel immunotherapies, such as CAR T cell therapy (CAR) and bispecific T cell engagers (BiTEs), are developed targeting different surface antigens, such as BCMA, SLAM7, CD38, or GPRC5D. Early use of immunotherapy may improve outcomes and several immunotherapy combinations have been recently approved for MM, and many others are under active investigation.

### Chimeric antigen receptor T (CAR-T)-cell therapy

Chimeric antigen receptor T (CAR-T) cells are engineered T cells that express a specific antigen (Ag) TCR that allows recognition of tumor Ag and killing of the cell. In CAR-T therapy, the patients’ T cells are selected from the peripheral blood, edited to express the chimeric antigen receptor, expanded, and reinfused into the patient^[122]^. The rapid activation and expansion of T cells, upon binding to target tumor Ag, can potentially lead to life-threatening complications such as cytokine release syndrome (CRS) and immune effector cell-associated neurotoxicity syndrome (ICANS)^[157]^. There are currently two FDA-approved CARs, both targeting BCMA that is highly expressed on malignant PCs: idecabtagene vicleucel (ide-cel/bb2121) and ciltacabtagene autoleucel (cilta-cel). A list of CAR T cell trials is summarized in Table 1. Ide-cel was the first CAR-T cell approved for patients with relapsed or refractory MM (RRMM), following the phase II KARMMA-1 trial results, showing an overall response rate (ORR) of 73%, median progression-free survival (PFS) of 8.6 months with 33% of patients achieving complete remission (CR)^[158,159]^. Recently, results from the phase 3 KARMMA-3 trial that recruited patients earlier in the disease course, after two to four previous lines, showed that at a median follow-up of 18.6 months, ORR was 71%, median PFS was 13.3 months, with overall survival data still immature^[160]^. Ide-cel is being tested in phase 1 KARMMA-4 trial in newly diagnosed multiple myeloma patients with high risk (R-ISS stage III), with the rationale that upfront use, where there may be more bone marrow reserve and less “exhausted” immune system, may offer an opportunity to replace transplant with CAR-T cell therapy. Cilta-cel was approved by the FDA for RRMM in February 2022 based on findings from phase I/II CARTITUDE-1 study, showing ORR of 97%, CR achieved in 82%, and 27-month PFS and OS rates of 55% and 70%, respectively^[161]^. Similar to ide-cel, ciltacel is also being tested upfront in phase 3 CARTITUDE-5 trial for NDMM who are non-transplant-eligible (NTE) and in phase 3 CARTITUDE-6 in NDMM who are transplant-eligible [Table 1].

Despite the high response rates, the majority of patients with MM exposed to CAR T targeting BCMA relapse within two years^[162]^. Several mechanisms of CAR-T cell resistance were speculated: (1) intra-tumoral BCMA expression heterogeneity leading to the selection of low-BCMA expressing clones; (2) T cell exhaustion; (3) cleavage of BCMA by gamma-secretase enzymes, which releases a soluble BCMA (sBCMA), acting as a decoy; and (4) activation-induced cell death (AICD) of the T cells^[163–165]^.

### Bispecific T-cell engagers (BiTEs)

Bispecific antibodies (BsAbs) are antibodies with two binding sites that recognize two antigens. Two types of constructs exist immunoglobulin (IgG)-like with an Fc fragment and non-Ig-like that lack the Fc fragment. Bispecific T-cell engagers (BiTEs) are a non-Ig-like subtype of BsAbs, consisting of two antigen recognition domains, one for CD3 of the TCR complex on T cells and one for the specific tumor antigen on the cancer cell. Through this interaction, BiTEs redirect autologous T cells in proximity to the tumor cells to facilitate T-cell activation, cytokine release, and killing of the cancer cell^[166]^. Unlike CAR-T cells, BiTEs are antibody-based molecules that are available off-the-shelf, which allows faster delivery as a treatment. The adverse events are similar to CAR-T cells, as they both activate the immune system leading to CRS and neurotoxicity, though less extensively^[167]^. The main target for MM BiTE studies is BCMA, with few studies investigating CD38, CD19, GPRC5D, and FcRH5. Table 2 summarizes trials for BiTEs. In October 2022, teclistamab (CD3 × BCMA) was approved for heavily treated RRMM, who have received at least 3 prior lines, following the results of phase I/II MajesTEC-1 trial that showed ORR 63%, 39% achieving CR, median PFS of 11 months. The most common toxicities were CRS (72%), neutropenia (71%), and infections (76%)^[168]^. A phase 3 trial was recently opened to investigate teclistamab alone versus pomalidomide, bortezomib, dexamethasone (PVd) versus carfilzomib, dexamethasone (Kd) in RRMM who have received one to three lines (NCT05572515).

Elranatamab is another anti-BCMA BsAb under investigation. In phase 1 MagnetisMM-1 trial, heavily pretreated RRMM received weekly subcutaneous elranatamab administration either alone, with lenalidomide or with pomalidomide. The ORR at the recommended phase 2 dose was 83%^[169]^. The updated follow-up data reported an ORR of 64%, with 31% of patients achieving CR. CRS was observed in 67% of patients with no grade 3 or higher CRS^[170]^. Overall, considering response rates and safety profile, elranatanab was granted breakthrough therapy designation by the FDA for the treatment of RRMM patients who have previously been treated with at least 4 prior lines of therapy^[170]^.

Characterization of the myeloma cancer cells has been instrumental in guiding the design of CARs. BiTEs targeting other MM surface antigens other than BCMA are being developed and have demonstrated promising results. G-protein coupled receptor family C group 5 member D (GPRC5D) is a recently identified molecule highly expressed on malignant MM plasma cells in the BM, but not on other healthy cells. In phase 1 MonumenTAL-1 trial, Talquetamab (CD3 × GPRC5D) was administered subcutaneously weekly, reaching ORR of 70% and the FDA granted it a breakthrough therapy designation in July 2022 for the treatment of RRMM who have received at least 4 prior lines^[171,172]^.

FcRH5 is a type 1 membrane protein that is almost uniquely expressed on B cells and plasma cells. Cevostamab (CD3 × FcRH5) has shown encouraging results in phase I study enrolling RRMM with an ORR of 54% at the 160 mg dose level in the expansion cohort.

CD38 is well known to the myeloma community as a surface molecule highly expressed in MM cells^[100]^. The clinical benefits of anti-CD38 mAb daratumumab led to studies engineering BiTEs targeting CD38. GBR 1342 (CD3 × CD38) is currently being investigated in a phase 1 trial for RRMM patients. Importantly, GBR 1342 specifically binds to a distinct epitope of CD38 and does not compete with daratumumab^[173]^.

As described above, numerous BiTEs trials are currently active and showing high response rates, with preliminary reports showing that BiTEs might be able to rescue the progression of disease in patients with advanced MM. The challenge that clinicians are encountering in real practice is (1) prevention of serious infections that can be deadly; (2) development of guidelines that direct clinicians on when to interrupt treatment with BiTEs; and (3) evaluating the safety of combination with other myeloma drugs.

### Antibody-drug conjugates (ADC)

Antibody-drug conjugates (ADCs) are monoclonal antibodies that are conjugated with highly cytotoxic compounds via a chemical linker. ADC binding to a tumor-associated antigen induces internalization of the cytotoxic agent and tumor cell death, resulting in theoretically diminished toxicity against normal cells. Belantamab mafodotin is the first-in-class ADC designed to bind to BCMA on plasma cells. The FDA approved belantamab for RRMM who had been previously heavily treated with at least four lines. Approval was granted following the results of the phase II DREAMM-2 trial, which showed an ORR of 31%, median PFS of 2.8 months, and median OS of 13.7 months^[174]^.

Several DREAMM studies are ongoing, as described in Table 3. Notably, the use of belantamab has resulted in frequent ophthalmologic toxicities such as corneal keratopathy (72%)^[174]^.

Clinical trials investigating ADCs targeting other antigens and utilizing other cytotoxic agents are currently ongoing. These include lorvotuzumab mertansine (anti-CD56 conjugated to cytotoxic maytansinoid) and indatuximab ravtansine (anti-CD138 conjugated to cytotoxic maytansinoid)^[165,175,176]^.

### Monoclonal antibodies (mAbs)

#### CD38 MAbs

CD38 is a glycoprotein that is highly expressed on malignant PCs but is also present at lower levels on normal PCs, myeloid and lymphoid cells, red blood cells, and platelets^[100]^. Because CD38 is not exclusive to MM cells, targeting CD38-positive cells can induce off-target NK depletion as well as favorable depletion of immunosuppressive Tregs and Bregs^[25,177,178]^. Daratumumab (Dara) was the first mAb approved by the FDA for the treatment of MM, initially only for RRMM patients and in recent years also for NDMM, both transplant-ineligible and transplant-eligible. The results of the main anti-CD38 trials are summarized in Table 4. Daratumumab was first approved as monotherapy on November 16, 2015, based on the results of the SIRIUS trial that enrolled MM patients who have received at least three prior lines of therapy (median PFS 3.7 months, ORR 29%)^[179]^. In the following years, trials were designed to combine daratumumab with PIs bortezomib/velcade (V) and carfilzomib/kyprolis (K) or IMiDs thalidomide (T), lenalidomide/revlimid (R) and pomalidomide (P) with the addition of steroids dexamethasone (d) or prednisone (P). In 2016, daratumumab was approved for RRMM who have received at least one prior line, following results of the CASTOR trial (Dara-Vd; median PFS of 16.7 months) and the POLLUX trial (Dara-Rd; median PFS of 44.5 months)^[180,181]^. Importantly especially for older patients, in May 2018, based on an ALCYONE trial, Dara was approved for non-transplant-eligible NDMM in combination with bortezomib, melphalan, and prednisone (Dara-VMP; median PFS of 36.4 months)^[182]^. Similarly, based on the MAIA trial, FDA approved Dara-Rd for non-transplant-eligible NDMM (median PFS of about 5 years)^[183]^. In September 2019, the FDA also approved Dara in the first-line setting for transplant-eligible NDMM based on data from the CASSIOPEIA study (Dara-VTd)^[184]^. Dara was also investigated in combination with carfilzomib instead of bortezomib in the APOLLO study, which led to the approval in 2021 of Dara-pomalidomide/carfilzomib for RRMM^[185]^.

Similar to daratumumab, isatuximab (isa) targets the CD38 receptor on a different epitope. The ICARIA-MM trial led to the approval of isa/pomalidomide/dexamethasone for RRMM (median PFS of 11.5 months)^[186]^. Similarly, the IKEMA study led to the approval of isa/carfilzomib/dexamethasone for RRMM (median PFS 35.7 months)^[187]^. Isatuximab is also being investigated upfront as part of the induction and consolidation regimens in the IsKia trial in transplant-eligible patients comparing Isa-KRd vs KRd (NCT04483739).

### SLAM7 MAbs

Elotuzumab (E) is a monoclonal antibody that targets the signaling lymphocytic activation molecule family member 7 (SLAM7). SLAM7 is expressed on both MM and NK cells and exerts antitumor activity by activating NK cells directly and via CD16-mediated antibody-dependent cellular cytotoxicity (ADCC)^[188]^. ADCC occurs when the Fc receptors on NK cells bind to the Fc portion of elotuzumab and release toxic enzymes that kill the tumor cell^[188]^. Importantly, unlike daratumumab, although SLAM7 is expressed on NK cells, elotuzumab does not induce NK cell fratricide^[188]^. On the contrary, SLAM7 was detected at high levels on exhausted CD8+ T cells and elotuzumab was able to specifically eliminate these cells^[189]^. In 2015, following the ELOQUENT-2 trial, the FDA approved elotuzumab/lenalidomide/dexamethasone for RRMM (PFS 19.4 months, ORR 79%)^[190]^. In 2018, following the ELOQUENT-3 trial, the FDA approved elotuzumab/pomalidomide/dexamethasone for RRMM (PFS 10.2 months, ORR 53%)^[191]^. Recently, a phase 1 clinical trial has investigated the use of elotuzumab in conjunction with peripheral blood cells to boost NK cell function in the early post-transplant setting, showing no concerns for safety^[192]^. However, it was found that the suppressive MM microenvironment actively inhibits the function of immune cells, including NK cells, leading eventually to relapse^[192]^.

## CONCLUSION

Multiple myeloma is a clinically and molecularly heterogenous disorder, which is typically characterized by immunoparesis, involving both innate and adaptive compartment, contributing to disease recurrence and therapy resistance^[193]^. The evolution from MGUS to SMM to active MM is associated with editing of the immune context. Unfortunately, most of these changes remain to be fully elucidated. As MM development is strictly supported by the interaction with the BM microenvironment, treatment targeting this cross-talk is necessary to eradicate this disorder. Over the years, several approaches aimed at evoking the immune response against MM have been developed, although none of them so far appeared to be as revolutionary and successful in MM as some of them are in other tumor models. Although the reason for this lack of response remains elusive, the profound exhaustion of the immune system might play a pivotal role. In other words, in MM, there is no identifiable dominant immunosuppressive mechanism that can be appointed as a key target to restore immune competence. Instead, MM is characterized by a complicated and redundant network of molecular and metabolic axes that can compensate for each others’ functions when targeted by immunotherapeutic strategies. A potential therapeutic strategy would be using immunotherapy early when the immune system is still intact, and there is a low tumor burden disease in order to harness patients own immune system to target the cancer cells and prevent progression.

In recent years, incredible advancement has been made in understanding the biology of MM clones and the immune microenvironment. Studies thus far have shown that the biology of the immune microenvironment is heterogenous and different between distinct patients and plastic within the same patient upon therapy. Multiparametric flow cytometry employing several markers holds great promise in incorporating a comprehensive characterization of the immune environment into the design of clinical trials. For example, patients that show at diagnosis absence of an “exhausted” immune system may benefit from immunotherapy, such as CAR-T and BiTEs, upfront. Given the immense availability of more effective and tailored treatments in the near future, clinicians and scientists will be able to design more personalized therapeutic plans for myeloma patients with unique microenvironments.

## Figures and Tables

**Table 1.** Summary of CAR-T cell clinical trials in MM.

Target	Trial name (Name of the drug) - phase	Study population	Outcomes	NCT
BCMA	KARMMA1 (idecel) – phase 2	RRMM	ORR 73%; CR 33%; mPFS 12.1 months – FDA approved	NCT03361748
BCMA	KARMMA3 – Phase 3	RRMM	ORR 71%; mPFS 13.3 months – FDA approved	NCT03651128
BCMA	KARMMA4 – Phase 1	High risk NDMM	NA	NCT04196491
BCMA	CARTITUDE1 (ciltacel) – Phase 1b/2	RRMM	ORR 97%; CR 82% – FDA approved	NCT04133636
BCMA	CARTITUDE5 – Phase 3	NTE NDMM	NA	NCT04923893
BCMA	CARTITUDE6-Phase 3	TE NDMM	NA	NCT05257083

RRMM, relapsed refractory multiple myeloma; NDMM, newly diagnosed multiple myeloma; ORR, overall response rate; mPFS, median progression free survival; CR, complete remission; NCT, national clinical trial

**Table 2.** Summary of BiTEs clinical trials in MM.

Target	Trial name (Name of the drug) - phase	Study population	Outcomes	NCT
BCMA × CD3	MajesTEC-1 (teclistamab) – phase 1/2	RRMM	ORR 63%; CR 39%; mPFS 11 months – FDA approved	NCT04557098
BCMA × CD3	MajesTEC-9 (teclistamab) – phase 1/2	RRMM	NA	NCT05572515
BCMA × CD3	MagnetisMM-1 (elratamab) – Phase 1	RRMM	ORR 64%; CR 31%	NCT03269136
CD3 × GPRC5D	MonumenTAL-1 trial, (talquetamab) – phase 1	RRMM	ORR 70%	NCT03399799
CD3 × FcRH5	Cevostamab	RRMM	ORR 54%	NCT03275103
CD3 × CD38	GBR 1342	RRMM	NA	NCT03309111

RRMM, relapsed refractory multiple myeloma; ORR, overall response rate; mPFS, median progression free survival; CR, complete remission; NCT, national clinical trial; NA, not applicable-data immature

**Table 3.** Summary of antibody-drug conjugates (ADC) clinical trials in MM.

Target	Trial name (Name of the drug) - phase	Study population	Outcomes	NCT
BCMA	DREAMM-2 (belantamab) – phase 2	RRMM	ORR 31%; mPFS 2.8 months, mOS 13.7 moths – FDA approved	NCT03525678
BCMA	DREAMM-3 (belantamab) – phase 3	RRMM	NA	NCT04162210
BCMA	DREAMM-8 (belantamab) – phase 3	RRMM	NA	NCT04484623
BCMA	DREAMM-9 (belantamab) – phase 1	NDMM	NA	NCT04091126

RRMM, relapsed refractory multiple myeloma; NDMM, newly diagnosed multiple myeloma; ORR, overall response rate; mPFS, median progression free survival; mOS, median overall survival; NCT, national clinical trial

**Table 4.** Clinical trials investigating monoclonal antibodies in MM.

Drug – target molecule	Trial name	Study population	Outcomes	NCT
Daratumumab – CD38	SIRIUS	RRMM	ORR 29%; mPFS 3.7 months	NCT01985126
	CASTOR	RRMM	ORR 83%; mPFS 16.7 months	NCT02136134
	POLLUX	RRMM	ORR 92.9%; mPFS 44.5 months	NCT02076009
	APOLLO	RRMM	mPFS 12.4 months	NCT03180736
	ALCYONE	NTE NDMM	ORR 90.9%; mPFS 36.9 months	NCT02195479
	MAIA	NTE NDMM	ORR 92.9% months; mPFS 5 years	NCT02252172
	CASSIOPEIA	TE NDMM	ORR 92.6% CR 39% months; CR 54%	NCT02541383
Isatuximab – CD38	ICARIA - MM	RRMM	ORR 60.4%; mPFS 11.5 months; mOS 24.6 months	NCT02990338
	IKEMA	RRMM	ORR 87% months	NCT03275285
	IsKia	TE NDMM	NA	NCT04483739
Elotuzumab – SLAM7	ELOQUENT-2	RRMM	PFS 19.4 months, ORR 79%	NCT01239797
	ELOQUENT-3	RRMM	PFS 10.2 months, ORR 53%	NCT02654132

RRMM, relapsed refractory multiple myeloma; NDMM, newly diagnosed multiple myeloma; TE, transplant eligible; NTE, non-transplant eligible; ORR, overall response rate; mPFS, median progression free survival; CR, complete remission; NCT, national clinical trial

## Data Availability

Not applicable.
